# Perceived Benefits of Magdalena Energy Healing Sessions: An Exploratory Study of Clients’ Perspectives

**DOI:** 10.3390/healthcare11233087

**Published:** 2023-12-02

**Authors:** Alvina D. Brueggemann, Angela U. Ekwonye

**Affiliations:** 1 Department of Holistic Health Studies, St. Catherine University, St. Paul, MN 55105, USA; 2 Department of Public Health, St. Catherine University, St. Paul, MN 55105, USA; amekwonye034@stkate.edu

**Keywords:** complementary and alternative medicine, Magdalena energy healing sessions, complementary healing, integrative healing, energy healing, physical health, mental health, spiritual health, social benefits, perceived benefits, qualitative

## Abstract

Background: Energy healing techniques are associated with many physical and mental benefits. A qualitative study was conducted to understand clients’ experiences of a new energy healing modality called Magdalena Energy Healing. Methods: Semi-structured qualitative interviews were conducted after clients experienced 60 min Magdalena energy healing session(s). Twenty-five adults participated in the study. All participants received Magdalena energy healing from a certified, trained practitioner. Thematic analysis was conducted to determine clients’ perceived benefits of the Magdalena energy healing session(s). Results: Four themes emerged from the data: Physical, Mental, Social, and Spiritual Benefits. Physical health benefits included relief from a variety of medical symptoms, improved sleep quality, and physical body awareness. Mental Health benefits included relaxation and peace, decision-making clarity, relief of mental health symptoms, and an increased ability to cope with life. Social Benefits included improved attitudes in relationships. Spiritual Benefits included optimism, gratitude, self-acceptance, and increased spiritual connection. Conclusions: Participants’ perceptions are that Magdalena energy healing sessions offer peace, symptom relief, and gratitude. Magdalena energy healing can address priorities of The National Center for Complementary and Integrative Health (NCCIH). Sessions can be seamlessly integrated into traditional medical care as a useful complementary/integrative healing option to improve physical, mental, and/or spiritual wellbeing across a variety of diseases.

## 1. Introduction

Complementary and integrative therapies are popular in the United States [1,2]. Approximately 38 percent of adults reported using complementary and alternative medicine (CAM), according to the National Health Interview Survey (NHIS) [3]. Energy healing, in particular, has gained popularity as a non-invasive and non-pharmacological approach [4,5]. Energy healing modalities are based on the understanding that all aspects of life, physical and non-physical, have an underlying subtle “energy” and that blockages in this energy system can lead to illness [6]. The energy system can impact the physical and emotional well-being of each person, and by working with the system at the energy level, one can promote well-being on all levels [7]. One energy healing modality that has not yet been studied is Magdalena energy healing, which is becoming popular worldwide. Certified Magdalena Energy Healing practitioners are offering sessions in the United States (US), Canada, Norway, Austria, Belgium, France, Ireland, Australia, New Zealand [8], and will soon be adding practitioners in Japan and Chile [9].

### 1.1. Effectiveness of Energy Healing Modalities

There are a variety of different energy healing modalities [10,11]. All energy therapies have the assumption that humans have subtle energies, or a life force, also called a ‘biofield’, and that disruptions to a naturally flowing energy system can lead to disease [10]. Some energy healing modalities employ a physical object near the client to influence the body’s energy field (e.g., gem/crystal therapy, flower essences). Other techniques use manual/hand pressure to influence the channels of subtle energy systems throughout the body (e.g., acupuncture, emotional freedom technique (EFT), reflexology). Other emotional healing modalities place hands near or lightly on the body to help focus energy healing (e.g., Reiki, healing touch, therapeutic touch, Qi Gong).

Reiki, healing touch, therapeutic touch, and Qi Gong all have a component where the practitioner focuses energy by imagining connection to a universal life energy source. Practitioners are not required to ascribe to a particular religion; they simply need to believe that there is a higher power or universal life energy that can be focused to heal clients. In Reiki, for instance, in one of the steps for advanced healers, practitioners can energetically and mentally connect to the positive qualities, attributes, and subtle energies of spiritual masters who have passed on, such as Jesus or Buddha [12]. In Magdalena energy healing sessions, similarly, practitioners are not required to practice a certain religion (Christianity); however, the spiritual attributes and qualities of Mary Magdalena, as a prominent female figure and spiritual role model, serve as a focal point for practitioners to focus healing energy to the client.

The effectiveness of energy healing has been demonstrated for various health conditions [13,14]. Mangione, Swengros, and Anderson [15] found that biofield therapies, such as healing touch and Reiki, increase relaxation, decrease anxiety and stress, and improve mood. Lee, Jeon, Huang, Cheon, and Ko [16] published an overview of systematic reviews indicating that Qi Gong and Tai Chi can decrease blood lipid level, reduce blood pressure, facilitate mobility, prevent falls, and improve overall quality of life. A study of the effects of Reiki on patients undergoing knee replacement surgery found that only the Reiki group showed significant reductions in pain, blood pressure, respiration rate, and state anxiety [17]. Another study found that a single session of Reiki improved pain, drowsiness, tiredness, nausea, appetite, shortness of breath, anxiety, depression, and overall well-being of participants [18]. A different study of hospitalized patients who received either a Reiki or massage session found improvements in pain, nausea, fatigue, anxiety, depression, and overall well-being [19,20]. Mixed results for energy therapies on healing rates have also been reported in some randomized controlled studies [21,22,23].

The research on Reiki and other energy healing modalities is mostly quantitative research on clinical effectiveness, but as we look to new energy healing modalities, it is important to understand subjective perceived experiences as well. As Broom [24] describes, “a qualitative interview-based study seeks to establish an in-depth understanding of the experiences of the respondents and the meanings within their accounts of a particular action, process or event.” Warber and colleagues stressed the importance of qualitative research in understanding energy healing modalities and in generating theories for further testing [7]. For example, a qualitative study of German spiritual healing noted that healers and clients described positive changes in perceived body sensations, increased well-being, positive emotions, and symptomatic relief of medical complaints [25].

This study focused on a new spiritual healing modality known as the Magdalena energy healing session(s), which is based on a female saint, Mary Magdalena. So far, there has been no research conducted on this new energy healing modality. This study explored clients’ perceived benefits of Magdalena energy healing sessions.

### 1.2. Magdalena Energy Sessions

According to personal communication with Mary Sise [26], Magdalena energy healing sessions originated in 2017 in upstate New York. Mary Sise, a social worker and spiritual teacher, under the guidance of her spiritual leader Sai Maa Lakshmi Devi, developed a new type of energy healing that focused on qualities embodied by a Christian saint: Mary Magdalena. The timing aligned with the Pope’s declaration, in 2016, that Mary Magdalena would be celebrated with a special feast day rather than the more typical memorial day for a saint. Mary Sise saw an opportunity to adapt an energy healing practice from her spiritual teacher, Sai Maa, to focus on the energies of this female saint. By learning about Mary Magdalena and her positive emotional qualities, practitioners would be able to tap into the ever-existing energy associated with her personality and to be able to focus that energy to heal clients. Mary Sise proposed the idea to her spiritual teacher, who agreed to ‘activate’ the energies. Sai Maa encouraged Mary Sise to train a group of practitioners to conduct this energy healing work. Mary Sise has been training a new cohort of students in this modality each year since 2017. The teaching team has since expanded to include 13 teachers who are training practitioners in Europe, North America, South America, and Japan. Since its inception, 168 trainees have become certified practitioners, and 111 have completed training. Upon completion of one year of training, trainees must be in good standing and pass a course on the ethics of energy work to be eligible to receive the initiation into the Magdalena frequencies. When these steps are complete, trainees are eligible for membership into the Magdalena Healing Society, a pre-requisite for receiving the activation and subsequently completing a practicum. The training is described further in the Materials and Methods section below.

A foundational principle in Magdalena healing training is that practitioners’ energy and emotional state can have an impact, positive or negative, on others, particularly clients, when they are offering a Magdalena energy session [27]. Magdalena energy healing is based on the premise that all subtle “energy”, particularly human thoughts and emotions, exists on a continuum of different frequencies. On the fear/contraction/separation end of the spectrum are emotions like terror, panic, blame, shame, guilt, judgment, and anger, while on the expansion/uplifting/love end of the spectrum are emotions like love, joy, compassion, clarity, tenderness, kindness, and gratitude. Magdalena healing considers the most expansive, uplifting frequencies to be the most promoting of healing, and practitioners must embody those frequencies, not merely contemplate them, to be successful. Much of the Magdalena energy healing training focuses on coaching practitioners in methods to shift, heal, or transmute lower energies (associated with negative emotions) within themselves to better hold and embody the high frequencies needed in the Magdalena energy healing sessions. Thus, practitioners must maintain a high state of energy (uplifting emotions) whenever offering Magdalena energy healing sessions.

A typical Magdalena energy healing session consists of a set appointed time between the practitioner and client where they meet in person in a quiet, often very sacred, space. Before the start of the session, the practitioner takes a few minutes to align themself with a spiritual Higher Self, using the centering technique: Activate, energize, and anchor the Light to invite the energy of Mary Magdalena. The practitioner welcomes the client and invites them to lie on a treatment table in a relaxed state or, in some instances, sit supported in a chair if that is more comfortable. Clients stay fully clothed, and the practitioner positions their hands at least six inches above the body and moves their hands over the body in a specific sequence, infusing the energetic layers around the body, the chakras, and organs with healing light. The energy work occurs in the client’s energy system (or subtle anatomy), including the energetic layers around the body (sometimes called the aura) and chakras. The client does not have to do anything special; they simply receive the energy. At the end of the session, the practitioner assists the client in descending from the table to a comfortable chair. The client is given water and can rest for a few minutes. A typical session lasts for about an hour, and sessions can be offered remotely or in-person.

For remote sessions, the practitioner notifies the client by phone or by email when the sessions will begin. The name, Magdalena healing session, is standardized across practitioners, and there is no standard scripting for how to introduce the session to a client; practitioners can focus on the logistics of the session and describe as much or as little about Mary Magdalena as makes sense for their practice and for the client. The client finds a comfortable spot to sit or lay down during the appointed time, in the client’s own home. The practitioner has a table set up in their own space, and the practitioner imagines the client on their table while doing the same physical movements/energy activations that they would do if the client was physically on their table. Distant healing has been described in other studies for practices such as Reiki, therapeutic touch, and reconnective therapy [28]. Often, the practitioner reaches out by phone or Zoom to let the client know when the session is complete and to check in with how the client is doing during and/or after the session.

For the Magdalena energy healing sessions, there have been anecdotal testimonies of benefits such as stress release, enhanced peacefulness, greater perception of meaning and purpose in life, and connection to the divine. Anecdotal experience suggests that Magdalena energy may help promote oneness, peace, devotion, and tenderness. Clients report feeling deep tenderness and love, calm, and relaxation. Since Magdalena energy healing is a new kind of energy work, we aim to conduct initial qualitative exploratory research to understand clients’ perceived benefits of Magdalena energy healing sessions.

## 2. Materials and Methods

### 2.1. Design

This was a phenomenological study to understand the experience of Magdalena energy healing from the perspective of the clients. Semi-structured interviews were conducted using Google Hangouts Meet and audio recorded with a dedicated audio recorder, separate from the video. This study was approved by the St Catherine University Institutional Review Board (IRB) Protocol #1513.

### 2.2. Recruitment Methods

Purposive sampling was used to recruit study participants from a listserv of people who have participated in Magdalena programs and/or healing sessions or have signed up for sessions at the Magdalena Healing website at https://magdalenahealing.com/. On behalf of the researchers, the Magdalena Healing organization sent out an email with the research announcement, which included a brief description of the purpose of the research, participation requirements, and the research inclusion/exclusion criteria. Interested clients were asked to email the researchers for more information. Participants were included if they had received at least one Magdalena energy healing session with a certified Magdalena practitioner and were fluent in English. To avoid a conflict of interest, we excluded participants who had donated to the Magdalena Healing organization research fund, Magdalena practitioners, or those training to become practitioners.

### 2.3. Participants

Twenty-five adults (eighteen females, six males, one nonbinary) between 25 and 65 years old (median age = 58) participated in the study. All of the participants received at least one Magdalena session from certified practitioners. Participant demographics are shown in Table 1.

### 2.4. Procedure

Researchers contacted potential participants to schedule a time for the interview. The researchers emailed participants a link to Google Hangouts Meet along with the consent form 48 h before the interview. At the beginning of the interview session, researchers ensured that participants had signed the consent form and/or obtained verbal consent. Participants then answered a series of demographic questions. An audio recording was started and the researchers asked the following interview questions: Did you notice any changes after your Magdalena energy healing session in your (a) Physical health, (b) Mental health, (c) Relationships, (d) Spiritual connection or spirituality? We conducted 25 in-depth interviews, and each interview session lasted for 40–50 min. Data were stored securely and kept confidential. Participant names were removed from transcripts prior to analysis. Participants were compensated with a choice of either a USD 15 Amazon gift card or a subsequent free Magdalena session for their time. The Amazon gift card was more frequently chosen. Twenty-one out of twenty-five participants (84%) chose to be compensated with the Amazon gift card.

### 2.5. Practitioner Qualification and Experience

All Magdalena energy healing practitioners in this study were certified Magdalena practitioners. Certified practitioners, at a minimum, undertake a 1-year training program to prepare them to receive an energetic initiation and to be able offer sessions to clients. Practitioners are not required to have any special religious affiliation or background. Training includes the following components [26]: (A) a commitment to a dedicated spiritual practice—a daily meditation which includes breathwork and visualization inviting in Mary Magdalena’s energy into oneself; (B) attendance at live and/or recorded trainings with 133 h of coursework across four modules: (1) self-healing through transmutation of lower frequencies and emotional awareness, (2) preparation for energetic initiation, (3) ethics and the science of energy work, and (4) teaching protocol [9]; and (C) participation in facilitated small groups. Once the year of training is complete, trainees must be in good standing and pass a course on ethics of energy work to be eligible to receive the initiation into the Magdalena frequencies. Trainees are eligible for membership into the Magdalena Healing Society, which is a pre-requisite for receiving the activation and subsequently completing a practicum. In the practicum, practitioners in training must provide at least 25 gifted (free) sessions to clients before becoming certified. All clients who receive these sessions provide anonymous feedback that is reviewed by the certification committee prior to certifying the practitioner. If any issues are noted in the feedback from session recipients, the committee can intervene and correct the issue, retraining the practitioner before certification. Once certification is complete, continuing education, including review of ethics curriculum, is required for maintenance of certification, with re-application required every 3 years. Additionally, to maintain certification, ethical complaints are investigated by the ethics committee, who determines how best to address the complaint. All Magdalena Energy Healers in this qualitative research had gone through training and successfully passed certification requirements [9].

### 2.6. Data Analysis

The researchers conducted a thematic analysis of the interview transcripts following the six steps described in Braun and Clarke [29]. First, in Step 1, the research team familiarized themselves with the data by reading and re-reading each transcript and agreed on topic codes. In Step 2, the team independently coded all responses to each interview question and agreed on 91% of the initial coding instances. They reviewed, discussed, and resolved the remaining differences in the coding. In Step 3, the team placed similar codes together to create potential themes. During this data analysis step, the team worked collaboratively and frequently had to revisit codes from Step 2 and the original data from Step 1. In Step 4, the research team looked for potential overlaps, similarities, and differences in how participants responded to each question. In Step 5, themes were defined and named. Throughout the data analysis process, the team went back and forth between the themes, categories, codes, and participant responses to ensure that the codes were accurately organized. The research team noted examples of participants’ responses under each theme. Finally, the authors included individual participant quotes as examples in this final report.

## 3. Results

Participants’ motivation to attend Magdalena energy healing session(s) was varied. Eleven participants attended the session because they were interested in the spiritual connection and wanted to experience and understand how Magdalena energy healing sessions work. Six participants were encouraged to go to the session by their family/friends. Another six went because the Magdalena Healing Society offered free sessions. Four participants reported being motivated to attend Magdalena sessions because they needed energy support. Two participants went because of a mental health condition with hope for a positive impact, and one participant was drawn to the session because the practice was consistent with their belief system. Participants in study interviews were recruited based on purposive sampling [30]. While this type of sampling can ensure that a wide audience is reached, one of the limitations is that it can lead to recruitment bias, as participants who are most interested in voicing their opinions can self-select to be in the study. In particular, participants who are most (or least) satisfied with their experiences may be more inclined to sign up for the study compared to those who had a neutral experience.

**Figure 1:**
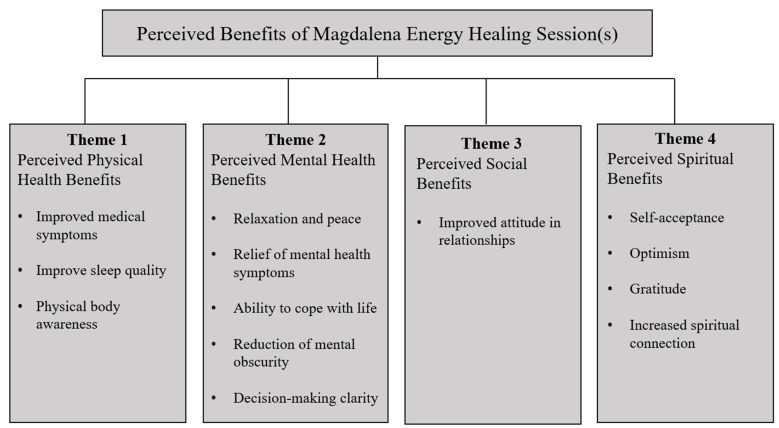
Perceived benefits of Magdalena energy healing sessions.

Participants described many benefits from the session(s), including physical, mental, social, and spiritual benefits. A summary of the themes and categories is displayed in Figure 1.

### 3.1. Theme 1: Perceived Physical Health Benefits

Perceived physical health benefits included improved medical symptoms, improved sleep quality, and increased body awareness.

#### 3.1.1. Improved Symptom Relief

Participants reported relief from medical symptoms. For instance, one participant described the experience of symptom relief from migraines:

*Prior to the session, I had some sort of a migraine, a little bit, and that had gone completely.* [Mag SC]

Another person described symptom relief from arthritis:

*With the cold weather that we’re having, my arthritis has been acting up and flaring up a little bit. I had a real sense of a layer falling off of the pain that I’ve been experiencing.* [Mag LB]

Symptom relief expanded beyond migraines and arthritis. For instance, one participant described the experience of abdominal relief with the following comment:

*My stomach tends to get bloated a lot. I’ve been struggling with all sorts of digestive issues. It’s like the light started to sink down in there. It wasn’t uncomfortable. It was like filtering into the heavy stuff in there. I felt lighter at the end of the session; it felt lighter in my abdominal area*. [Mag BH]

Another participant described a change in clarity of vision after experiencing the Magdalena session:

*I’d like to have my vision return to 20/20 vision. What I did notice was there was, I’ll say, almost like a film over my eyes. I felt like I was always looking through a veil, and that is gone. So there’s much more clarity within my vision*. [Mag SC]

In addition to vision, migraines, arthritis, and digestive relief, multiple participants experienced knee/hip pain relief, as exemplified by the following comment:

*I had been having a pain in my hip for about two months and it was really severe and I thought, I might need a hip replacement. And she didn’t know about that at the time she was doing remote work on me and within about 30 min, the pain completely disappeared and it never came back.* [Mag SB]

In summary, participants reported symptom relief for migraines, arthritis, digestion, vision, and knee/hip pain.

#### 3.1.2. Improved Sleep Quality

The Magdalena energy healing sessions seemed to have helped improve the sleep quality of a few participants.

*I had my session at eight, between eight and eight thirty at night. So after that, I just went under the covers and slept, you know, until 3:30 in the morning? I felt totally rested. I felt perfectly good.* [Mag SC]

*I had this session in the evening, so I fell asleep right into the night. And so that I woke up the next morning feeling well rested for the first time in years, it was huge, like, oh my God, wow.* [Mag CN]

#### 3.1.3. Physical Body Awareness

The journey toward self-awareness begins with physical body awareness. Physical body awareness is the ability to recognize early signs the body may express [31]. A few participants described how they became more aware of what is happening to their physical body.

*I get a sense of where my body is now, where my feet are, what sensations I’m having, and then energetically, I could feel a sensation of breath happening through each zone.* [Mag LS]

*I felt much more aggressive when I ate meat. I became aware of the effect of meat on my aggressiveness; my aggressiveness was fading slowly after I had the Magdalena sessions. So then I eat less meat and then my aggressivity, I can feel it’s less.* [Mag BS]

### 3.2. Theme 2: Perceived Mental Health Benefits

Clients reported numerous mental health benefits such as relaxation/peace, relief of mental health symptoms, an increased ability to cope with life, reduction in mental obscurity, and clarity in decision making.

#### 3.2.1. Relaxation and Peace

Participants reported feelings of relaxation and inner peace. The following quotes capture these sentiments.

*I generally felt peaceful after it, you know, my mind felt very clear and peaceful.* [Mag BH]

*I was just like peaceful and open and relaxed and drift, you know, floating, you know, allowing my thoughts to come and go.* [Mag PR]

#### 3.2.2. Relief of Mental Health Symptoms

Mental health conditions can be experienced by youth, adults, and older adults. Mental health illnesses include schizophrenia, anxiety, depression, autism spectrum disorder, and more [32]. According to some participants in this study, Magdalena energy healing sessions improved their mental health symptoms. One participant described how anxiety and depression appeared physically in her body, and how her healing session was able to unblock the associated energy, leading to a more positive mental state:

*I get a lot of anxiety and depression throughout my life and it feels like sometimes a tight ball in my solar plexus… It [the Magdalena energy session] unblocks the heavy sludge that I feel inside my body, energetically just kind of moves things around and I just feel lighter, more relaxed, just more in my body. It’s really hard to describe to somebody that’s not used to having energy work.* [Mag SB]

Another participant noticed a significant change in his subjective experience but also felt that Magdalena energy healing sessions changed his brain chemistry:

*And there was a time in my life, up to the age of 20, where opening a book was not that difficult for me, but after various traumatic experiences and two major year long depressive episodes and anxiety, it’s just learning the staying up and being able to stay up paying attention. That has gone away: the Magdalena session helped not only shift the state, the chemistry, and the wiring but it’s also helped me stack a new structure in my physical brain.* [Mag ZC]

Another common mental health condition is Autism Spectrum Disorder (ASD). ASD is a complex developmental condition involving persistent challenges with social communication, restricted interests, and repetitive behavior [33]. A participant mentioned that Magdalena energy healing sessions might be helpful for people with Autism Spectrum Disorder:

*In my honest opinion, I really do think that Magdalena would’ve helped someone on the spectrum. Because someone on the spectrum processes their world completely different. I’ve noticed that someone not on the spectrum, it’s as if they were pre-made prepared to interact with their environment very easily. Whereas someone on the spectrum it’s not necessarily because they had traumas, but the way they can convert their thoughts, emotion, energy, and feelings into the world around them. So from mind to actions, it can be a challenge for them. The Magdalena really does help process everything.* [Mag ZC]

Another participant described how the Magdalena session helped her overcome food addiction:

*Yeah, so things were really like in my face my food issues, it became really apparent that I’ve been addicted to sugar and wheat. I was very heavy in myself, very lethargic. So then the week after that [Magdalena energy healing session] I actually stopped eating sugar. Like, I was really consuming like a packet of biscuits every night. And I’ve just not done that now for about a week.* [Mag SL]

#### 3.2.3. Ability to Cope with Life

Participants noted a significant change in their ability to deal effectively with difficult situations in their lives.

*[I was] able to navigate a stressful situation that happened once I got home from orientation that I wasn’t expecting; I was pretty calm through it all.* [Mag LB2]

*And there’s a divorce going on right now and I’ve been trying to keep it amicable, but you know, there’s two people here and it was really dramatic the weekend before, and amicable had fallen apart. And so I was processing all of that and the Magdalena session kind of flushed it all out. And then, I’m back into being able to be stronger and heal in a whole new space. It was amazing*. [Mag CN]

#### 3.2.4. Reduction in Mental Obscurity

Some participants noticed that their minds shifted from a more obscure state to a more clear state after the Magdalena energy healing session(s).

*I had a real sense of just being so clear, like my whole field before we started, it was a little bit staticky feeling because of the pain that I was experiencing and the inflammation. But afterwards, it was clarity at all levels, mind, body, and spirit. I just felt an incredible, like crystal clear sense and feeling.* [Mag LB]

*I think that sometimes I have this kind of like brain fog, you know, and I have trouble figuring stuff out or focusing, and it also could be kind of a stress thing too. I don’t know. But, I would say that was gone. So like, it was just easier to kind of process things that were going on around me. Just like, I can come up with words faster, sort of like remember names a little bit better … and kind of remembering timelines better.* [Mag BH]

#### 3.2.5. Decision-Making Clarity

Participants were able to identify priorities and make clearer decisions:

*I mean, there’s never a way I could do everything, so there’s always, what am I going to drop. I was having a hard time figuring out how to get space for my practice, but after the Magdalena sessions, I knew what I needed to do. It’s getting clearer what I am choosing to do.* [Mag CN]

*With regards to my business and my career, there’s still some things that I have questions on, but my next step is clearer to me. Like I said, a clarity of what I needed to do and the decisions I needed to make in my business, because right now I have a couple of options or a couple different things that I could do and I always wasn’t doing either one of them and I was like waiting. After the sessions I could now see what I can do, and then trusting that the rest of it will work out.* [Mag BK]

### 3.3. Theme 3: Perceived Social Benefits

Participants noticed changes in their social interactions after the Magdalena energy healing session(s). In particular, participants experienced a positive shift in attitude towards family and friends.

Improved Attitude in Relationships

Participants noted an increase in kindness, less judgment, and more compassion towards others.

*I’m much nicer to my husband. And I’m much nicer to me. I have a kinder voice that, to me, that inner dialogue is kinder. … it’s more gentle, it’s more loving, really it’s more loving. [I’m] more able to listen more because I’m more quiet in my body.* [Mag SC]

*My roommate, for instance, did something last week that really triggered me and I was feeling pretty upset at him and the energy work really helps a lot with that type of stuff to just not be as upset and to just kind of pull back a little bit and just not be so judgmental towards him I guess.* [Mag SB]

One participant described the experience of feeling compassionate in the midst of a difficult life situation:

*My sister has had a long journey with bipolar and struggles with that … and she checked herself into the hospital. And so that’s always upsetting, but I just went and I felt a lot of compassion for her and I just felt peaceful and I felt good about my boundaries … It sounds like a big change too, in some ways that was a positive change after the Magdalena session.* [Mag LB]

### 3.4. Theme 4: Perceived Spiritual Benefits

Perceived spiritual benefits included self-acceptance, optimism, gratitude, and an increased spiritual connection.

#### 3.4.1. Self-Acceptance

Self-acceptance is the concept that a person will be loved regardless of what they say, think, or do, positive or negative. If a person does something wrong, they can forgive themselves even in the face of failures. People can also appreciate the good qualities about themselves while expressing self-love. Magdalena energy healing sessions helped participants experience more self-forgiveness, self-acceptance, and self-love.

*I’m probably more forgiving of myself and that’s a nice place to be also.* [Mag JJ]

*So yeah, I do notice a difference and you know, just in my daily life I’m a dancer and I take dance classes and I do a little bit of teaching. And when I first started dancing, I was so judgmental of myself, constantly criticizing myself and now I have so much fun. And when I make a mistake, I laugh at it. It’s just the self-acceptance part. It’s just a lovely gift to get from this kind of work.* [Mag SB]

The same participant described how connecting to a higher power can allow someone to experience self-love:

*You gotta love yourself. Cuz what does that really mean? … When you really experience that coming from something bigger than you … I can open up to that and I can let those forces heal me*. [Mag SB]

#### 3.4.2. Optimism

A few participants of the Magdalena energy healing sessions were more optimistic and hopeful about their future with these words.

*I felt like some sort of certainty, that everything’s gonna be okay kind of feeling, you know?* [Mag EB]

*More of a trust that everything is for my best and trusting that it is working out and knowing that.* [Mag BK]

#### 3.4.3. Gratitude

Gratitude was expressed by many participants in this study. One participant recounted how the experience of the Magdalena energy healing session improved their sense of gratitude, forgiveness, and concern for the common good.

*I would say forgiveness, but it’s more than that. It’s forgiveness and love of humanity. Once you understand it is for the greater good, you are able to forgive. I was able to forgive everyone and be at, like, so lower vibration [negative emotions] cannot affect you when you are filled with gratitude and all the stuff like that. Gratitude and love for your life and stuff.* [Mag BS]

A different participant connected the experience of gratitude with divinity:

*There’s a feeling of gratitude like a limitless expansion of something that is so wonderful. It’s a deep sensation of being filled from the divine and it’s just like, oh my God, this is just so good for my heart, for my soul.* [Mag SC]

#### 3.4.4. Increased Spiritual Connection

Participants reported a stronger connection with spiritual practices and/or divine figures.

*I was so much focused on this life– on having to take care of everything, having to eat, having to smoke a cigarette, having to do whatever to feel good, but now I’m reminded that if I connect to my spirit and my soul, and if I ask for connection to other spirits, then, and—I’m getting chills now—then I’m being touched. And then I can feel this heart connection, and I can do that whenever.* [Mag EMK]

*I definitely felt like there were angels or some kind of higher beings that were holding me in light. And I just feel like I’m more tuned into that since that session. Oh, I’ve been able to get to profound places since then and quite quickly.* [Mag SB]

One participant has increased her daily spiritual practice to commune more with spiritual guides (Mary Magdalena) and spiritual teachers (Sai Maa).

*Well, I guess I could say what I notice is more of an intentionality around connecting with Mary Magdalena every day. Like intentionally being like: ‘good morning, Mary Magdalena, thank you for being here today for supporting me,’ like intentionally naming her, looking at her picture on my wall, where maybe before that, it wasn’t as daily intentional. So, and maybe the same thing, like with Sai Maa; doing that with Sai Maa. So I’d say those two things I noticed.* [Mag MS]

In summary, four themes emerged from the data: Physical, Mental, Social, and Spiritual benefits.

## 4. Discussion

The efficacy of complementary modalities has been demonstrated for various health conditions [13,14]. Unlike other energy healing modalities, Magdalena energy healing is a new modality that activates the masculine and feminine energies through the powerful healing frequencies of Mary Magdalena. Based on the premise that all “subtle energy”, particularly human thoughts and emotions, exists on a continuum of different frequencies, Magdalena practitioners embody those frequencies and infuse the energetic layers with healing light into their clients.

Participants described the physical, mental, social, and spiritual benefits they gained from participating in the sessions. These benefits mirror the biopsychosocial–spiritual model of care. According to this model, the biological, psychological, social, and spiritual dimensions are distinct dimensions of a person. No one aspect can be separated from the whole since each can interact and affect other aspects of the person [34]. It seems that Magdalena energy healing sessions affected the totality of participants’ relational existence—physical, psychological, social, and spiritual. This is consistent with many ancient healing traditions, which have the belief that wellness exists when there is a balance of the physical, mental, social, and spiritual components of our being [18,31].

Regarding the physical health benefits, participants noted a decrease in a variety of medical symptoms. While energy healing should always be considered as an integrative and/or complementary part of a patient’s regimen, it was interesting to note how participants observed the lessening or disappearance of symptoms such as migraines, arthritis, digestive discomfort, visual film, and hip/knee/back pain after experiencing the Magdalena energy healing session. Other energy healing modalities have been associated with improvement in physical and/or medical symptoms [17,19,20]. Improved sleep was another benefit that clients experienced after the Magdalena session. Many reported sleeping extremely well or deeply following the energy healing exchange. This might have occurred because Magdalena energy healing promotes energetic unblocking, where the person starts to perceive themselves as being calmer, as there is a relaxation of body muscles. An improvement in sleep quality was also observed in a sample of nurses who participated in weekly Reiki sessions [35].

Participants also reported improved physical body awareness. Increased body awareness was demonstrated through participants’ descriptions of increased kinesthetic knowledge of their body parts and/or breath. Paying attention to the body leads to learning the body’s wisdom and with this knowledge comes the power to be in greater control of knowing the best choice to help the body regain or move towards balanced health. Increased body awareness has been associated with improved body image, prevention of relapse in substance use disorders, and less trauma dissociation [36,37,38] of emotional states, such as aggression or increased food cravings. This awareness can lead to better physical and mental health.

Mental health benefits included a sense of relaxation and peace, relief of mental health symptoms, an increased ability to cope with life, reduction in mental obscurity, and decision-making clarity. A majority of participants in this study reported feeling more peaceful or relaxed after receiving a Magdalena energy healing session. In fact, across energy healing modalities, participants reported an increased sense of peace and stress relief [39].

Participants also commented that Magdalena energy healing sessions helped release negative feelings associated with anxiety and depression. Other studies have demonstrated the effectiveness of energy therapies in improving anxiety and depression symptoms among clients [20,40]. While traditional therapy can involve a significant cognitive load for the client, in the case of Magdalena energy healing, participants noted a sense of ease in being able to sit back and have the energy unblock their systems without conscious effort. It was also worth noting that one participant hypothesized that Magdalena energy sessions had, over time, ‘rewired’ his brain, leading to significant changes in being able to process the world around him. The participant also speculated that Magdalena energy sessions could be useful for people “on the spectrum”, or people who may have Autism Spectrum Disorder (ASD). There is evidence that people with ASD tend to have an absence of emotional responsiveness in neurotypical situations [41]. This participant postulated that the translation of thought, energy, and feelings to the world was much easier after having experienced Magdalena energy healing sessions, and that energy healing could benefit many autistic individuals.

Participants also described the ability to cope with difficult life situations, such as a divorce or an unanticipated event. Participants described being more calm and emotionally regulated. Coping can be problem-focused or emotion-focused [42]. Problem-focused coping aims to change the actual event, whereas emotion-focused coping focuses on one’s interpretation of the event. In this study, participants engaged in emotion-focused coping and had an expanded perspective on their problems/relationships, as well as an increased sense of calm and balanced emotions when faced with unanticipated or challenging events.

A few participants also experienced increased clarity in thought and decision making, which is less commonly reported in the literature. Participants were able to remain calm and prioritize among many options. One participant had enough clarity and courage to pursue a career she had been putting off for twenty years. This improved decisiveness could have come about as a result of less stress, more relaxation, and greater awareness of self. The increased clarity described by participants in this study is consistent with those reported for other energy-healing modalities [43].

Social Benefits from Magdalena energy healing sessions included more kindness, less judgment, and more compassion in relationships. When experiencing an upsetting event, participants responded with less distress and attachment. Their emotional responses then included compassion, setting boundaries, and accepting the other person. One mechanism for improved relationships may be self-compassion, which includes (1) the ability to self-soothe in response to distress, (2) an emphasis on common humanity that promotes a sense of connection rather than isolation, and (3) a balanced and non-judgmental response to negative emotions, as opposed to avoiding or becoming overwhelmed by them [44]. An expression of kindness and compassion was even found to increase bonding between individuals of different generations [45].

Spiritual Benefits included self-acceptance, optimism, gratitude, and increased spiritual connection. Participants learned not only how to extend compassion and acceptance to others in relationships, but also to themselves. Magdalena energy healing sessions helped participants experience more self-forgiveness, self-acceptance, and self-love. Self-acceptance can propel an individual on the journey towards a more balanced body, mind, and spirit [31].

Participants also reported feeling optimistic and that things would work out positively for their benefit. Greater optimism is associated with physical and psychological well-being [46,47], giving participants a potentially protective effect on their mental and physical health. Gratitude is also a protective factor in mental health [48]. Participants experienced gratitude as an overflowing sense of thankfulness. As spiritual awareness heightens, one begins to feel thankful for the ordinary things in life. Some participants noted that the gratitude they felt was springing from a divine, spiritual source of connection and that it led to a feeling of connection with humanity. Gratitude allows the vicious cycle of negative emotions to be replaced with positive ones and ultimately contributes to the feeling of abundance [31]. Finally, participants reported a deeper, more intentional spiritual connection. They described tuning in to the qualities and/or frequencies of divine beings more often and feeling joy in that connection with angels, spirits, and spiritual teachers. While some tuned in specifically to the energy of Mary Magdalena, others described connecting with a wider variety of spiritual practices. The Magdalena energy healing session(s) left many participants feeling accepting of themselves, optimistic, grateful, and more connected with spirit.

### 4.1. Implications

While there are a number of traditional techniques, such as medication and therapy, to help with mental illness and physical symptoms, it is imperative to have a variety of options for treatment. Magdalena energy healing may be helpful as complementary/integrative support for those who are suffering from a mental or physical illness. Magdalena energy healing sessions can offer a non-invasive approach that is gentle and does not require substantial cognitive burden. It can also offer the option of relief and support for a diverse population who may or may not be comfortable with verbal modes of treatment. Participants who have a strong connection to spirituality may also prefer this energy healing modality, given its connection to a particular spiritual figure, Mary Magdalena. Recommendations on how to integrate complementary/integrative approaches into primary care can be found in a variety of sources [49,50,51].

### 4.2. Limitations

A purposive sample was used in this study, which limits the generalizability of the findings. Purposive sampling can lead to recruitment bias, where participants who are most interested in voicing their opinions (for instance, those who were most or least satisfied with their experiences) can self-select to be in the study [30]. In addition, only participants with a primary language of English were included in the study, and future studies could include participants who speak a variety of different primary languages. As a qualitative study, these are anecdotal, subjective experiences of clients, and we cannot determine unique causality. While certain participants experienced physical, mental, social, and spiritual benefits, others reported no change in their daily experiences after Magdalena energy healing sessions. The benefits could be due to Magdalena healing sessions, or due to other factors in clients’ lives. Randomized controlled trials should be conducted to determine the efficacy and/or effectiveness of the Magdalena energy healing sessions on physical, mental, social, and spiritual health.

## 5. Conclusions

In this qualitative research study, participants reported a variety of benefits from Magdalena energy healing sessions, including improvements in physical health, mental health, relationships, and spiritual connection. The presence of spiritual benefits is distinct from what we have seen in other modalities. More research should be conducted to determine the efficacy and effectiveness of Magdalena healing energy sessions.

## Figures and Tables

**Table 1:** Participant Demographics.

Demographics	n (%)
Gender	
Female	18 (72)
Male	6 (24)
Nonbinary	1 (4)
Race/Ethnicity	
White	21 (84)
Black	1 (4)
Asian	1 (4)
Mixed	1 (4)
Other	1 (4)
Education/Degree	
Primary–8th grade	1 (4)
High School/GED	4 (16)
Associate	1 (4)
Bachelor’s	12 (48)
Master’s	6 (24)
Professional	1 (4)
Age	
Median (Range)	58 (25–65)
Residence	
United States	18 (72)
Canada	3 (12)
United Kingdom	2 (8)
Other (S Africa, Portugal)	2 (8)
Occupation	
Social Services	9 (36)
Unemployed or Retired	8 (32)
Business/Finance	6 (24)
Other	4 (16)
Primary Language	
English	22 (88)
French	2 (8)
German	1 (4)
Religious/Spiritual Affiliation	
Spiritual	8 (32)
Open/multiple	4 (16)
None	4 (16)
Energy healing	2 (8)
Buddhist	2 (8)
Christian	2 (8)
Jewish	1 (4)

## Data Availability

The data are not publicly available due to IRB regulations including data security and participant confidentiality.
